# Discretionary decisions and disparities in receiving drug-eluting stents under a universal healthcare system: A population-based study

**DOI:** 10.1371/journal.pone.0179127

**Published:** 2017-06-08

**Authors:** Raymond N. Kuo, Chao-Lun Lai, Yi-Chun Yeh, Mei-Shu Lai

**Affiliations:** 1 Institute of Health Policy and Management, College of Public Health, National Taiwan University, Taipei City, Taiwan; 2 Division of Cardiology, National Taiwan University Hospital Hsin-Chu Branch, Hsin-Chu City, Taiwan; 3 Institute of Epidemiology and Preventive Medicine, College of Public Health, National Taiwan University, Taipei City, Taiwan; Universita degli Studi di Napoli Federico II, ITALY

## Abstract

**Objectives:**

One of the main objectives behind the expansion of insurance coverage is to eliminate disparities in health and healthcare. However, researchers have not yet fully elucidated the reasons for disparities in the use of high-cost treatments among patients of different occupations. Furthermore, it remains unknown whether discretionary decisions made at the hospital level have an impact on the administration of high-cost interventions in a universal healthcare system. This study investigated the adoption of drug-eluting stents (DES) versus bare metal-stents (BMS) among patients in different occupations and income levels, with the aim of gauging the degree to which the inclination of health providers toward treatment options could affect treatment choices at the patient-level within a universal healthcare system.

**Design and participants:**

We adopted a cross-sectional observational study design using hierarchical modeling in conjunction with the population-based National Health Insurance database of Taiwan. Patients who received either a BMS or a DES between 2007 and 2010 were included in the study.

**Results:**

During the period of study, 42,124 patients received a BMS (65.3%) and 22,376 received DES (34.7%). Patients who were physicians or the family members of physicians were far more likely to receive DES (OR: 3.18, CI: 2.38–4.23) than were patients who were neither physicians nor in other high-status jobs (employers, other medical professions, or public service). Similarly, patients in the top 5% income bracket had a higher probability of receiving a DES (OR: 2.23, CI: 2.06–2.47, p < .001), than were patients in the lowest income bracket. After controlling for patient-level factors, the inclination of hospitals (proportion of DES>50% or between 25% and 50%) was shown to be strongly associated with the selection of DESs (OR: 3.64 CI: 3.24–4.09 and OR: 2.16, CI: 2.01–2.33, respectively).

**Conclusions:**

Even under the universal healthcare system in Taiwan, socioeconomic disparities in the use of high-cost services remain widespread. Differences in the care received by patients of lower socioeconomic status may be due to the discretionary decisions of healthcare providers.

## Introduction

It has been reported that patients who receive a drug-eluting stent (DES) rather than a bare-metal stent (BMS) are less susceptible to restenosis and repeat revascularization [1, 2]. The clinical advantages of DES have led to its rapid replacement of BMS throughout the world. One year after the Food and Drug Administration approved DES (2005), a study using data from the U.S. National Cardiovascular Data CathPCI Registry found that more than 96% of the patients who fulfill “on-label” indications for DES elected to receive DES [3]. However, the higher price of DES may influence the patient’s choice of treatment. Studies have shown that the DES use is influenced by demographics, socioeconomic status, hospital characteristics, and the type of insurance [3–8]. Although the literature shows that the use of a DES is associated with socioeconomic status, most previous studies have used aggregated socioeconomic measures at a regional or community level as a proxy for individual patients [7, 8]. Furthermore, researchers have largely overlooked the relationship between the occupation of patients and disparities in the use of DES, despite the fact that the literature shows a clear link between a patient's occupation and their access to, benefits from, and experience with health care [9–14].

The inclination of healthcare providers toward a given mode of treatment may be another factor contributing to variations in the use of DES. Studies have shown that the decisions of physicians vary according to the local practice environment, even when dealing with patients presenting the same clinical conditions. However, most previous studies have measured the practice patterns of physicians using vignettes rather than ‘‘real-world” measures, such as chart reviews and/or direct observation [15].

Prior to December 2006, only BMS was covered by Taiwan’s National Health Insurance (NHI). The NIH now covers the cost of DES (all brands and types of stents) by providing a fixed reimbursement equal to that of a BMS. Thus, patients receiving DES are required to pay the difference out of their own pocket. Furthermore, hospitals in Taiwan are able to set their own price for DES, as a means of offsetting high supply costs in order to meet revenue targets. This raises the question of whether such a reimbursement scheme discriminates against patients from a lower socioeconomic status, and whether a hospital's inclination toward DES influences the adoption of DES at the patient-level. In this study, we used hierarchical modeling in conjunction with the population-based NHI claims database to examine the issue of DES use among patients in different occupations and income levels. Our aim was to investigate the inclination of health care providers with regard to treatment options and elucidate its association with the actual selection of treatment options at the patient-level.

## Materials and methods

### Data sources

This study used data from the population-based National Health Insurance database (NHID) for the period 2002–2010. This involved the collection of patient IDs, dates of the ambulatory or inpatient care provided, disease classification codes (i.e. the ICD-9-CM codes), physician IDs, physician specialties, hospital IDs, surgical and non-surgical procedures performed, and the medications prescribed for each case. We also used the National Health Insurance Enrolment file (within the NHID) to determine the socioeconomic status of the patients, including their occupation and income level. The IDs of all patients, physicians, and hospitals were encrypted to enable the cross-linking of data without compromising privacy. The release of all data was approved by the Department of Statistics under Taiwan’s Ministry of Health and Welfare. The protocol for this study was approved by the institutional review board (IRB) of the National Taiwan University Hospital (protocol # 201205016RIC).

### Study population and variables

This study included all patients who received either a BMS or DES between January 1, 2007 and December 31, 2010 and filed a claim with the NHI. For patients who were admitted more than once to receive a stent during this period, only the first admission was included in the study. Patients that had received a stent between 2002 and 2006 were excluded. In so doing we were able to exclude cases that involve revascularization as well as cases in which the patient opted for BMS or DES based on their satisfaction with a previous treatment. Patients that received both BMS and DES during the same admission period were also excluded. Patients whose socioeconomic status could not be identified from the enrolment file were also excluded from our analysis.

The types of occupation analyzed in this study included employers, public servants, physicians, other medical professionals, farmers, fishers, employees in other fields, and the self-employed. The patients’ (or insured’s) monthly, salary-based income was categorized according to the percentile of the general population: > 95% (>USD$2227), 75% to 95% (USD$1010—USD$2227), 50% - 75% (USD$640—USD$1010), 25% - 50% (USD$550—USD$640), above low income but less than 25%, and low income (government approved). For patients who were an unemployed spouse or relative, data of the insured individual was used. A hospital's inclination toward the use of DES was derived by dividing the total number of DES recipients by the total number of coronary stent recipients. The control variables included age, gender, comorbidities, and the year of admission. This study used the ICD codes reported by Quan et al. to determine whether the patient was diagnosed with selected comorbidities [16]. Morbidity was based on any diagnosis associated with an inpatient claim or any diagnosis that appears on at least two outpatient claims listed among the insurance claims of a patient one year prior to the index admission. All morbidity groups were treated as dichotomous variables. The comorbidities included in the models were first selected using multivariate logistic regression with stepwise selection.

### Statistical analysis

Hierarchical generalized linear modeling (HGLM) with the log link function was used to estimate the odds ratios (OR) that various patient groups would receive a DES, and to estimate the degree to which this was associated with hospitals’ inclination toward the implantation of DESs. Hierarchical models were used for the clustering of patients within hospitals while taking into account the random effects imposed by individual hospitals. The ‘Maximum Likelihood with Laplace Approximation’ method was used to estimate the parameters for each model. We adopted three models in this study: 1) controlled for age, gender, comorbidity, and year of admission, and 2) controlled for the above as well as employment status, occupation, and income class, and 3) controlled for the above as well as the proportion of patients who received a DES in the hospital to which they were admitted. We selected employees in other fields and the self-employed, as well as patients whose monthly salary was in the 25% - 50% percentile of the general population, as reference groups in the models. The fact that these groups are representative of the majority of the population and include a larger number of patients than in the other groups makes cross-group comparisons more straightforward. The performance of the models was compared according to their C-statistic (i.e., the area under the receiver operating characteristic (ROC) curve). The level of statistical significance was set at p < 0.05. All statistical operations were performed using SAS (version 9.4, SAS Institution Inc.).

## Results

The total number of cases of stent placement identified in the NHID data between 2007 and 2010 was 79 748. In this four-year observation period, we included only the first admission for a stent placement for a given patient (n = 69 742). From the total, we excluded individuals who had received a stent prior to 2007 (n = 3440) and those who had received a BMS as well as a DES within the same admission period (n = 2304). We also excluded 165 patients due to an inability to identify their socioeconomic status from the enrolment file (see S1 Fig). This resulted in the inclusion of a total of 64 500 patients (92.5%) in the study, as follows: BMS (42 124 patients) (65.3%) and DES (22 376) (34.7%).

**Fig 1:**
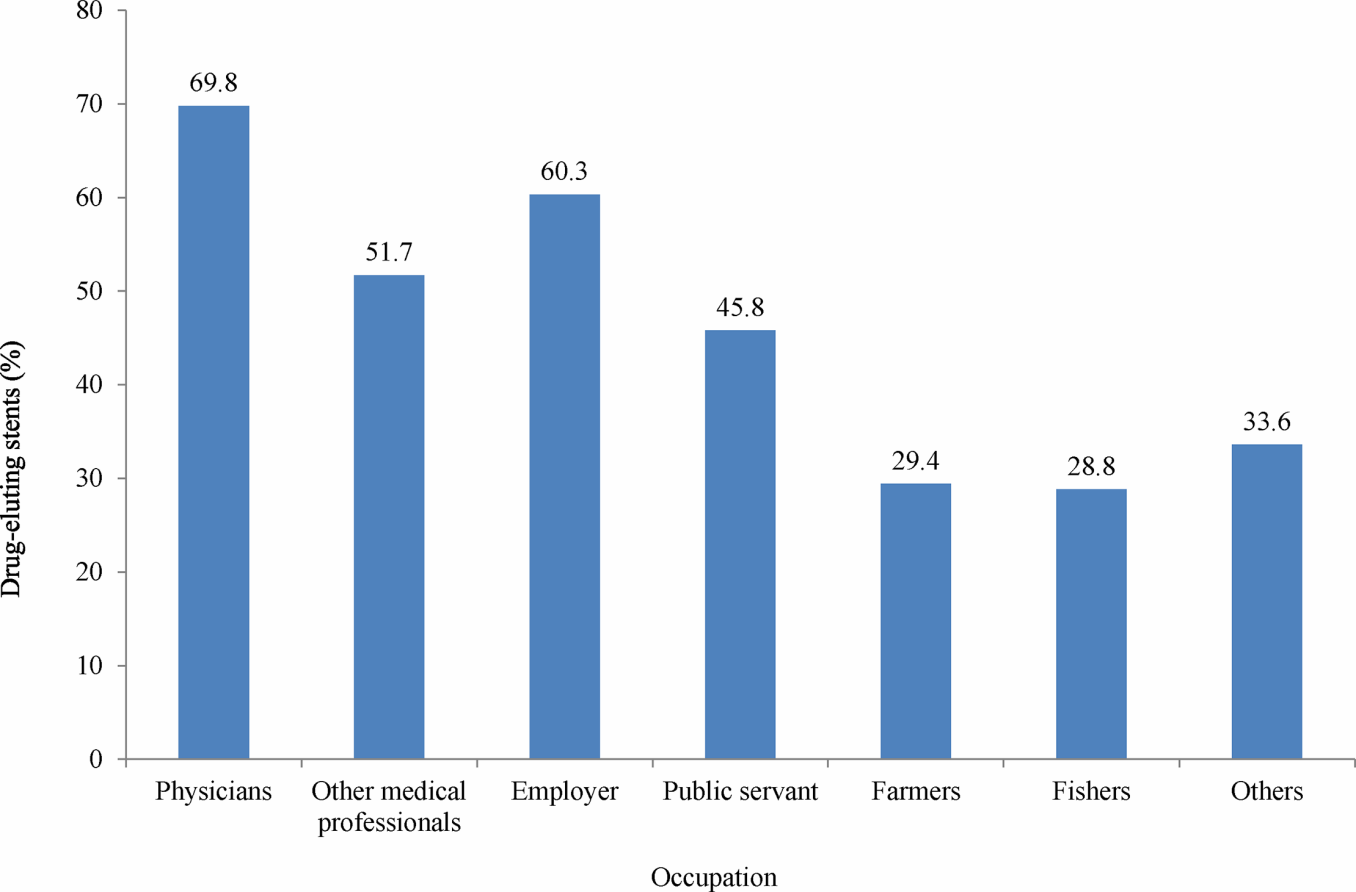
Proportion of patients received DES, by occupation.

**Fig 2:**
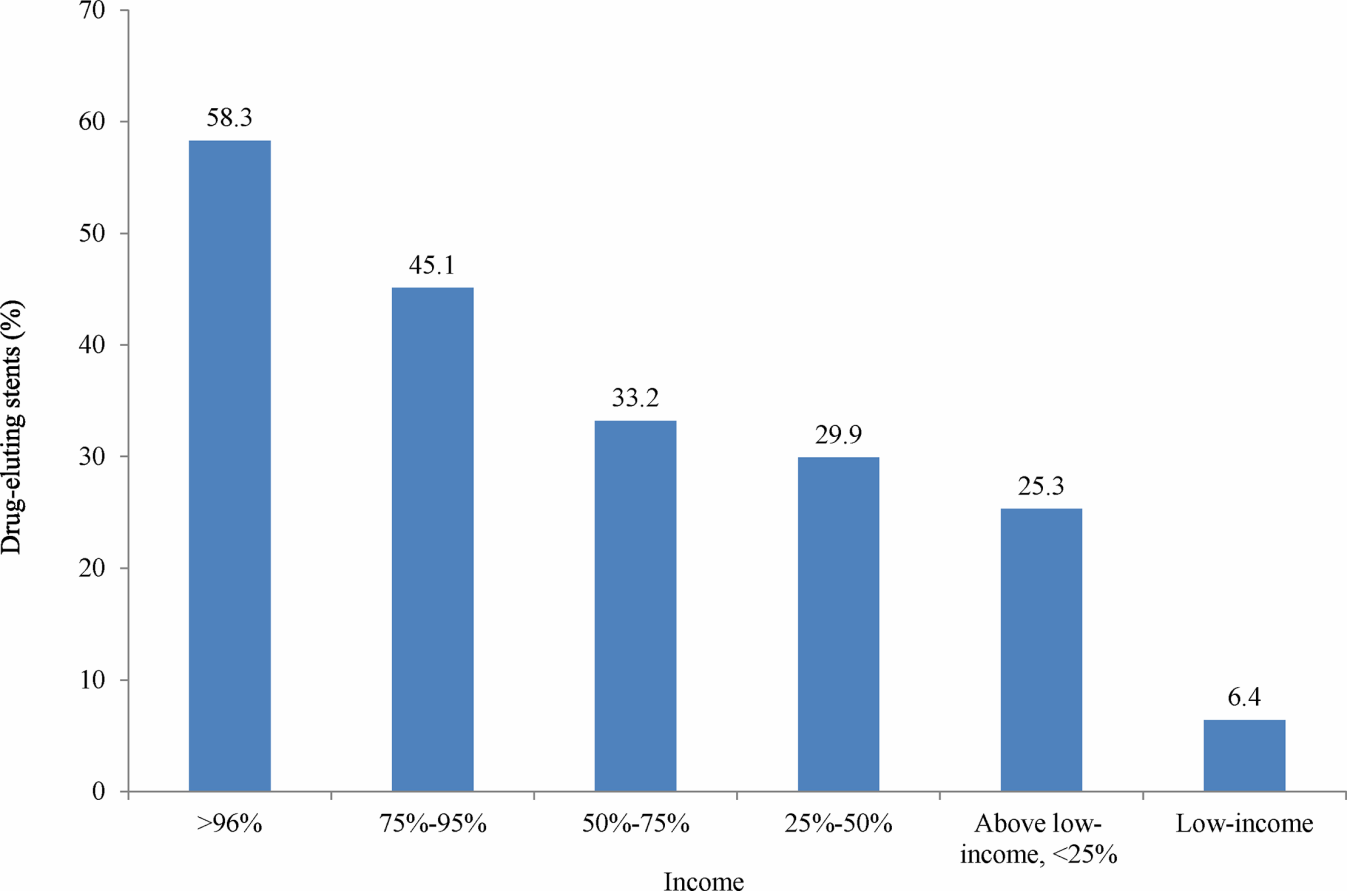
Proportion of patients received DES, by income.

As shown in **Table 1**, the average age of patients in this study was 65.4 years. Patients who received a DES were younger (p < .001) and had fewer comorbidities than did the patients who received a BMS. BMS procedures are fully covered by the NHI; however, DES procedures are partially covered by the NHI, such that patients are expected to pay the cost difference out of their own pocket. The proportion of employers, doctors, other medical professionals, and public servants in the DES group was higher than in the BMS group (p < .001). A higher proportion of patients who received a DES were in a higher income bracket (p < .001). **Fig 1**shows that more than half of the patients who were doctors, employers, and other medical professionals received a DES. In comparison, less than thirty percent of the fishers and farmers received a DES. As shown in **Fig 2,** income was positively associated with the likelihood of receiving a DES; i.e., 58% of the patients in the highest income bracket received a DES. In contrast, less than one-third of those below the 50 percentile received a DES.

**Table 1:** Distribution of patient characteristics: Patients who received a drug-eluting stent or bare-metal stent.

	Total (n = 64 500)		Bare-metal stent (n = 42 124)		Drug-eluting stent (n = 22 376)		p-value
	n	(%)	n	(%)	n	(%)	
Age							
Mean (S.D)	65.4	(12.4)	65.8	(12.7)	64.7	(11.7)	< .001
Elderly (> = 70 yr)	26 541	(41.2)	18 122	(43.0)	8 419	(37.6)	< .001
Gender							.009
Male	47 490	(73.6)	30 876	(73.3)	16 614	(74.3)	
Female	17 010	(26.4)	11 248	(26.7)	5 762	(25.8)	
Year of treatment							
2007	13 516		9 265	(68.6)	4 251	(31.5)	
2008	15 757		10 595	(67.2)	5 162	(32.8)	
2009	17 364		11 258	(64.8)	6 106	(35.2)	
2010	17 863		11 006	(61.6)	6 857	(38.4)	
Comorbidity							
Congestive heart failure	9 467	(14.7)	6 699	(15.9)	2 768	(12.4)	< .001
Cardiac arrhythmias	6 902	(10.7)	4 495	(10.7)	2 407	(10.8)	.736
Hypertension, uncomplicated	32 919	(51.0)	20 963	(49.8)	11 956	(53.4)	< .001
Hypertension, complicated	16 954	(26.3)	11 159	(26.5)	5 795	(25.9)	.104
Paralysis	596	(0.9)	439	(1.0)	157	(0.7)	< .001
Other neurological disorders	1 465	(2.3)	1 062	(2.5)	403	(1.8)	< .001
Chronic pulmonary disease	8 563	(13.3)	6 007	(14.3)	2 556	(11.4)	< .001
Diabetes, uncomplicated	21 269	(33.0)	13 695	(32.5)	7 574	(33.8)	.001
Hypothyroidism	503	(0.8)	299	(0.7)	204	(0.9)	.006
Renal failure	6 621	(10.3)	4 827	(11.5)	1 794	(8.0)	< .001
Metastatic cancer	241	(0.4)	189	(0.4)	52	(0.2)	< .001
Solid tumor without metastasis	2 645	(4.1)	1 816	(4.3)	829	(3.7)	< .001
Fluid and electrolyte disorders	1 864	(2.9)	1 443	(3.4)	421	(1.9)	< .001
Blood loss anemia	293	(0.5)	229	(0.5)	64	(0.3)	< .001
Deficiency anemia	525	(0.8)	394	(0.9)	131	(0.6)	< .001
Psychoses	240	(0.4)	191	(0.5)	49	(0.2)	< .001
Depression	1 988	(3.1)	1 287	(3.1)	701	(3.1)	.588
Employed	45 521	(70.6)	29738	(70.6)	15783	(70.5)	.872
unemployed spouse or relative	18 979	(29.4)	12386	(29.4)	6593	(29.5)	
Occupation							< .001
Employer	2 388	(3.7)	948	(2.3)	1 440	(6.4)	
Doctors	265	(0.4)	80	(0.2)	185	(0.8)	
Other medical professionals	464	(0.7)	224	(0.5)	240	(1.1)	
Public servants	4 382	(6.8)	2 375	(5.6)	2 007	(9.0)	
Farmers	13 823	(21.4)	9 754	(23.2)	4 069	(18.2)	
Fishers	1 403	(2.2)	999	(2.4)	404	(1.8)	
Employees or self-employed	41 775	(64.8)	27 744	(65.9)	14 031	(62.7)	
Income							< .001
>96%	2 884	(4.5)	1 204	(2.9)	1 680	(7.5)	
75%-95%	13 173	(20.4)	7 236	(17.2)	5 937	(26.5)	
50%-75%	10 657	(16.5)	7 118	(16.9)	3 539	(15.8)	
25%-50%	20 994	(32.6)	14 715	(34.9)	6 279	(28.1)	
Above low-income, <25%	1 322	(25.3)	11 411	(27.1)	4 911	(22.0)	
Low-income	470	(0.7)	440	(1.0)	30	(0.1)	

Table 2 shows that the proportion of patients in the same occupation or income class who received a drug-eluting stent differed considerably according to the hospital's inclination toward DES use (i.e., the proportion of patients in a given hospital who received a DES, compared to other treatment options) (both p < .001). Among the occupation subgroups, doctors were most likely to receive a DES: low-DES hospitals (54.4%) and high-DES hospitals (83.6%). Fishers were the least likely to receive a DES: low-DES hospitals (12.4%) and high-DES hospitals (55.5%). A similar trend was observed in the income subgroups. Those in the top five percentile were the most likely to receive a DES: low-DES hospitals (32.3%) and high-DES hospitals (81.9%). Those in the lowest income group were least likely to receive a DES: low-DES hospitals (12.1%) and high-DES hospitals (57.6). The results in **Table 2**reveal a strong correlation between the inclination of hospitals to implant a DES and the actual incidence of implanting the devices.

**Table 2:** Proportion of patients in the same occupation or income class who received a drug-eluting stent, as differentiated by the hospital's inclination toward DES use (i.e., the proportion of patients that received a DES compared to other treatment options).

	Hospital's proportion of patients receiving a DES								
	Low (< 25%, 24 hospitals)			Medium (25%—<50%, 49 hospitals)			High (> = 50%, 19 hospitals)		
	(n)	(%)	Received a DES (%)	(n)	(%)	Received a DES (%)	(n)	(%)	Received a DES (%)
All cases	20 127	100.0	16.3	33 409	100.0	36.2	10 964	100.0	64.1
Occupation^a^									
Employer	464	2.3	33.6	1 270	3.8	58.8	654	6.0	82.1
Doctors	57	0.3	54.4	135	0.4	68.9	73	0.7	83.6
Other medical professionals	118	0.6	38.2	244	0.7	48.4	102	0.9	75.5
Public servants	1 275	6.5	23.5	2 212	6.6	47.5	895	8.2	73.4
Farmers	5 728	28.5	15.4	6 689	20.0	34.5	1 406	12.8	62.6
Fishers	523	2.6	12.4	752	2.3	35.6	128	1.2	55.5
Employees or self-employed	11 962	59.4	15.0	22 107	66.2	33.9	7 706	70.3	61.5
Income^b^									
96%-	566	2.8	32.3	1 463	4.4	54.5	855	7.8	81.9
75%-95%	3 431	17.1	23.2	6 813	20.4	45.4	2 929	26.7	70.1
50%-75%	3 231	16.1	15.3	5 612	16.8	34.6	1 814	16.6	61.0
25%-50%	8 059	40.0	15.1	10 392	31.1	33.9	2 543	23.2	60.5
<25%	4 840	24.1	12.1	9 129	27.3	29.9	2 823	25.8	57.6

a. x^2^ = 1432.5, p < .001

b. x^2^ = 1440.4, p < .001

As shown in **Table 3**, elderly patients were less likely to receive a DES, whereas patients diagnosed with cardiac arrhythmia, hypertension, uncomplicated diabetes, or hypothyroidism were more likely to receive a DES. Patients who received stents in later stages of the study period were also more likely to receive a DES. The results of Model 3 show that after controlling for age, gender, and comorbidities, individuals who worked as an employer (OR: 2.04, p < .001) or doctor (OR: 3.18, p < .001) or other medical professional (OR: 1.82, p < .001), public servant (OR: 1.32, p < .001), or farmer (OR: 1.07, p < .001) had a higher probability of receiving a DES. Patients in the top income bracket were more likely to receive a DES (OR: 2.23, p < .001), than were those in the lowest income bracket (OR: 0.16, p < .001). Compared to the results of Model 2, the parameters of patient characteristics estimated using Model 3 were similar. The inclination of a hospital toward the use of DES is calculated by dividing the total number of DES recipients by the total number of coronary stent recipients at that facility. Our results show that this is strongly associated with the use of DES at the patient-level.

**Table 3:** Hierarchical regression models for estimating the probability of receiving a drug-eluting stent.

	Model 1				Model 2				Model 3			
	OR	95% CI (LL, UL)		p-value	OR	95% CI (LL, UL)		p-value	OR	95% CI (LL, UL)		p-value
Age												
Elderly (> = 70 yr)	0.82	(0.79	, 0.86)	< .001	0.88	0.84	0.92	< .001	0.88	(0.84	, 0.92)	< .001
Gender												
Male	0.96	(0.92	, 1.00)	.077	0.89	0.86	0.93	< .001	0.89	(0.85	, 0.93)	< .001
Comorbidity												
Congestive heart failure	0.83	(0.79	, 0.88)	< .001	0.85	0.81	0.90	< .001	0.85	(0.81	, 0.90)	< .001
Cardiac arrhythmias	1.13	(1.06	, 1.19)	< .001	1.13	1.07	1.20	< .001	1.13	(1.07	, 1.20)	< .001
Hypertension, uncomplicated	1.18	(1.14	, 1.23)	< .001	1.19	1.14	1.23	< .001	1.19	(1.14	, 1.23)	< .001
Hypertension, complicated	1.17	(1.12	, 1.22)	< .001	1.16	1.11	1.21	< .001	1.16	(1.11	, 1.21)	< .001
Paralysis	0.73	(0.60	, 0.88)	.001	0.74	0.60	0.90	.003	0.73	(0.60	, 0.89)	.002
Other neurological disorders	0.79	(0.70	, 0.90)	.000	0.80	0.71	0.91	< .001	0.81	(0.71	, 0.92)	.001
Chronic pulmonary disease	0.85	(0.80	, 0.90)	< .001	0.88	0.83	0.93	< .001	0.88	(0.83	, 0.93)	< .001
Diabetes, uncomplicated	1.10	(1.06	, 1.14)	< .001	1.12	1.07	1.16	1.12	1.11	(1.07	, 1.16)	< .001
Hypothyroidism	1.24	(1.02	, 1.50)	.033	1.24	1.02	1.51	1.24	1.25	(1.03	, 1.52)	.027
Renal failure	0.72	(0.67	, 0.76)	< .001	0.74	0.69	0.78	< .001	0.73	(0.69	, 0.78)	< .001
Metastatic cancer	0.57	(0.41	, 0.80)	.001	0.60	0.43	0.84	.003	0.61	(0.44	, 0.86)	.004
Solid tumor without metastasis	0.86	(0.78	, 0.94)	.001	0.85	0.78	0.94	< .001	0.85	(0.78	, 0.94)	.001
Fluid and electrolyte disorders	0.70	(0.62	, 0.79)	< .001	0.72	0.64	0.81	< .001	0.71	(0.63	, 0.80)	< .001
Blood loss anemia	0.59	(0.44	, 0.79)	.000	0.59	0.44	0.80	< .001	0.60	(0.44	, 0.81)	.001
Deficiency anemia	0.74	(0.59	, 0.91)	.005	0.71	0.58	0.89	.003	0.72	(0.58	, 0.89)	.003
Psychoses	0.56	(0.40	, 0.78)	.001	0.63	0.45	0.88	.007	0.62	(0.44	, 0.87)	.005
Depression	1.08	(0.98	, 1.20)	.138	1.09	0.99	1.21	.086	1.10	(0.99	, 1.22)	.070
Year of admission												
2008	1.08	(1.03	, 1.14)	.004	1.08	1.02	1.14	< .001	1.00	(0.95	, 1.06)	.960
2009	1.21	(1.14	, 1.27)	< .001	1.20	1.14	1.26	< .001	1.09	(1.03	, 1.15)	< .001
2010	1.44	(1.37	, 1.52)	< .001	1.44	1.37	1.52	< .001	1.16	(1.10	, 1.23)	< .001
Employed (vs. unemployed families)					1.16	1.11	1.21	< .001	1.16	(1.11	, 1.21)	< .001
Occupation												
Employer					2.03	1.85	2.24	< .001	2.04	(1.86	, 2.25)	< .001
Doctors					3.16	2.38	4.21	< .001	3.18	(2.38	, 4.23)	< .001
Other medical professionals					1.81	1.48	2.21	< .001	1.82	(1.49	, 2.22)	< .001
Public servants					1.34	1.25	1.44	< .001	1.34	(1.24	, 1.44)	< .001
Farmers					1.07	1.01	1.14	.025	1.07	(1.01	, 1.14)	< .001
Fishers					0.96	0.85	1.10	.565	0.96	(0.85	, 1.10)	.772
Employees or self-employed (reference)												
Income												
96%-					2.25	2.05	2.47	< .001	2.23	(2.06	, 2.47)	< .001
75%-95%					1.43	1.36	1.51	< .001	1.43	(1.36	, 1.51)	< .001
25%-75% (reference)												
<25%, above low-income					0.88	0.84	0.93	< .001	0.88	(0.84	, 0.93)	< .001
Low-income					0.16	0.11	0.23	< .001	0.16	(0.11	, 0.23)	< .001
Proportion of DES												
>50%									3.64	(3.24	, 4.09)	< .001
25–50%									2.16	(2.01	, 2.33)	< .001
<25% (reference)												
C-statistics		.712			.729					.734		
-2 Log Likelihood	75058.91				73493.94				72960.71			
AIC	75106.91				73563.94				73034.71			
BIC	75167.43				73652.21				73128.01			

OR: Odds ratio; CI: confidence interval; LL: lower limit; UL: upper limit.

## Discussion

Our analysis revealed the effects of socioeconomic indicators on the likelihood of receiving drug-eluting stents. Hierarchical regression analysis indicates that after controlling for patients’ age, gender and comorbidity, individuals who were physicians, employers, other medical professionals, and public servants were more likely to receive a DES. Correspondingly, individuals in lower income classes were less likely to receive a DES. We also found that the preference of hospitals toward the administration of DESs is strongly associated with actual implementation, which implies that this situation might depend on discretionary decisions made by healthcare providers.

In previous studies, occupation, income, and other patient-level socioeconomic characteristics were shown to be closely associated with access to, benefits from, and experience with health care [9]. This study adds to a growing body of literature addressing disparities in access to healthcare among patients from different socioeconomic backgrounds; i.e., treatment decisions are based on factors other than the clinical characteristics of each case. All of our findings except those pertaining to farmers are in good agreement with those of Hannan et al., who pointed out that this disparity existed in the past when the use of stents was rare as well as today when most patients (93%) receive a DES. Furthermore, they reported that this disparity remained even after controlling for hospital volume and tendency toward the use of a DES [5]. Yong et al. found that individuals with a low income are less likely to be administered a DES, which may be due to the fact that high-income patients are more likely to have insurance covering more costly procedures as well as subsequent dual antiplatelet therapy [8]. Recent studies have reported that patients with private insurance are more likely to receive a DES than are those with no health insurance and those who depend on public insurance schemes [17, 18]. A systematic review reveals that the association between socioeconomic status (SES) and access to treatment for coronary heart disease (CHD) was stronger when SES was measured based on individual-level compared to area level. This review also found that the association between SES and access to treatment for CHD was stronger for individuals living in countries without universal health coverage [19]. However, in this study, the cost of all of the stents (regardless of the brand or type) is covered by the NHI at a fixed value equal to the reimbursement cost for a BMS. This means that all patients have much greater access to stent implants. Nonetheless, the socioeconomic disparity remains.

We also found that patients who are more knowledgeable with regard to innovations in medicine (e.g. physicians and other medical professionals) were more likely to receive a DES. This finding is consistent with previous research indicating that people with more knowledge, money, power, prestige, and beneficial social connections are better able to avoid risk and adopt protective strategies [20]. Thus, it is conceivable that the advent of new technologies could widen socioeconomic disparities in health [21]. The price of DES may be an important factor contributing to the disparities observed in this study; however, it remains unclear whether other financial concerns affect the decisions of physicians or patients with regard to the use of DES. For example, the ability of a patient to adhere to antiplatelet therapy for the full 12 months duration may be a concern [19, 20]. During the study period, the NHI reimbursed patients for antiplatelet therapy over a period of three months, regardless of whether they received DES or BMS. Nonetheless, patients requiring antiplatelet therapy for an extended period would have to pay for it out of their own pocket, unless they met a set of specified cardiac conditions. The observed socioeconomic disparities may also be shaped by the financial incentives provided by the payment scheme. The profit margins for DESs are set by each hospital, and the financial return on DESs is generally better than that of BMS; therefore, it is conceivable that hospitals would encourage cardiologists to use DESs.

Our findings also revealed that the preference of hospitals for DESs is strongly associated with the actual implementation of DESs at the patient-level. A number of studies have reported that physicians in more affluent regions tend to use more resource-intensive interventions [22–24]. Other studies have reported on the discretionary power of physicians in the selection of cardiovascular interventions [15, 25, 26]. However, to the best of our knowledge, this is the first study to investigate discretionary decisions pertaining to the use of DESs. Surprisingly, based on the odds estimated using the hierarchical models, we found that the preference of hospitals for DES use has a stronger association than any patient characteristic on the actual selection of DES at the patient level. We also observed variations in the use of DESs among patients who were physicians or other medical professionals. It is likely that those patients are more aware of the difference between the two types of stent. This group would also be expected to seek a second opinion before making a decision. In this situation, the proportion of DES use should not vary among hospitals, regardless of the financial inclinations of the institution. However, our results indicate that DES use varies considerably among hospitals as well. Physicians presumably leave to the patient the decision of whether to receive a BMS or a DES; however, it is unclear why variability at the hospital-level is as great as it is. If the inclination of a hospital toward DES use were not a critical factor, then the proportion of patients receiving a DES should be the same among all patients with similar socioeconomic status, even among patients in low-income groups. It would therefore follow that differences in the use of DESs could be attributed primarily to socio-economic status and the preferences of patients. Accordingly, we would not observe a notable difference in DES use among patients with similar socio-economic status. If patient-level characteristics (e.g. income and occupation) are unable to provide a complete explanation for the variations in stent selection, then other hospital-level factors must be taken into account.

This study also revealed that physicians in highly pro-DES hospitals are more likely to recommend a DES to elderly patients. Some studies have reported that DESs have many advantages over BMSs when applied to elderly patients [27, 28]; however, one recent study reported that the outcomes of primary percutaneous coronary intervention (PCI) are worse for elderly ST Segment Elevation Myocardial Infarction (STEMI) patients than for non-elderly patients with the same condition. Another study revealed that elderly patients face a higher risk of mortality and bleeding (compared to younger patients), even when undergoing new-generation DES [29]. In an investigation into variations in the use of cardioverter-defibrillators (ICDs), Matlock et al. found that physicians in regions of higher ICD use were more likely to recommend an ICD to frail patients and patients with a short life expectancy–those who are less likely to benefit from cardiac interventions due to an increased risk of death from competing morbidities [15]. These findings are consistent with previous assertions that variations in clinical interventions are more pronounced when decisions are discretionary [22, 30].

Our analysis has a number of limitations that should be addressed. First, cardiologists will prevent implanting a DES into patients who are expected to undergo a scheduled surgery before they can complete the dual antiplatelet therapy. However, we were unable to determine from the claims data whether that was a concern for patients who received a BMS. Second, although the National Health Insurance claim data used in this study contain all inpatient, outpatient, and prescription claims for each patient, the decision to implant a DES may be based on factors that are not included in the data. Previous studies exploring discretionary decisions have commonly used clinical vignette responses to measure the tendency of physicians to implement particular interventions. However, this approach cannot be used to determine whether the responses are an accurate representation of the physician’s practice patterns in the real world [22]. Third, we found that the preference of hospitals for DES use has a greater influence than any patient characteristic with regard to the actual choice of DES at the patient level. Nonetheless, this situation would be greatly clarified if the variations in DES use could be attributed to specific factors. Researchers have devised alternative approaches to obtaining pseudo R-squares for generalized linear mixed-effects models [31]; however, the common statistical packages, such as SAS, do not include this option. Finally, patient preferences were not included in our analysis, and it must be remembered that the decisions of physicians may be influenced directly by the expectations and demands of patients, particularly when interventions are discretionary [22].

## Conclusions

This study found that the income and occupation of patients as well as the inclination of hospitals toward DES are all associated with the actual adoption of DES at the patient level. The National Health Insurance benefits plan greatly increases access to DES; however, socioeconomic disparities remain. The preference of hospitals with regard to DES use was shown to be strongly associated with the patients’ decision to receive a DES. Our results highlight the importance of investigating the effects of discretionary decisions, particularly when financial incentives may be involved.

## Supporting information

S1 Fig Sample selection flowchart. (TIF)
