# Supplementary figures and images for: Discretionary decisions and disparities in receiving drug-eluting stents under a universal healthcare system: A population-based study
Source: PLoS One. 2017 Jun 8;12(6):e0179127. doi: 10.1371/journal.pone.0179127 (PMC5464647; doi:10.1371/journal.pone.0179127)

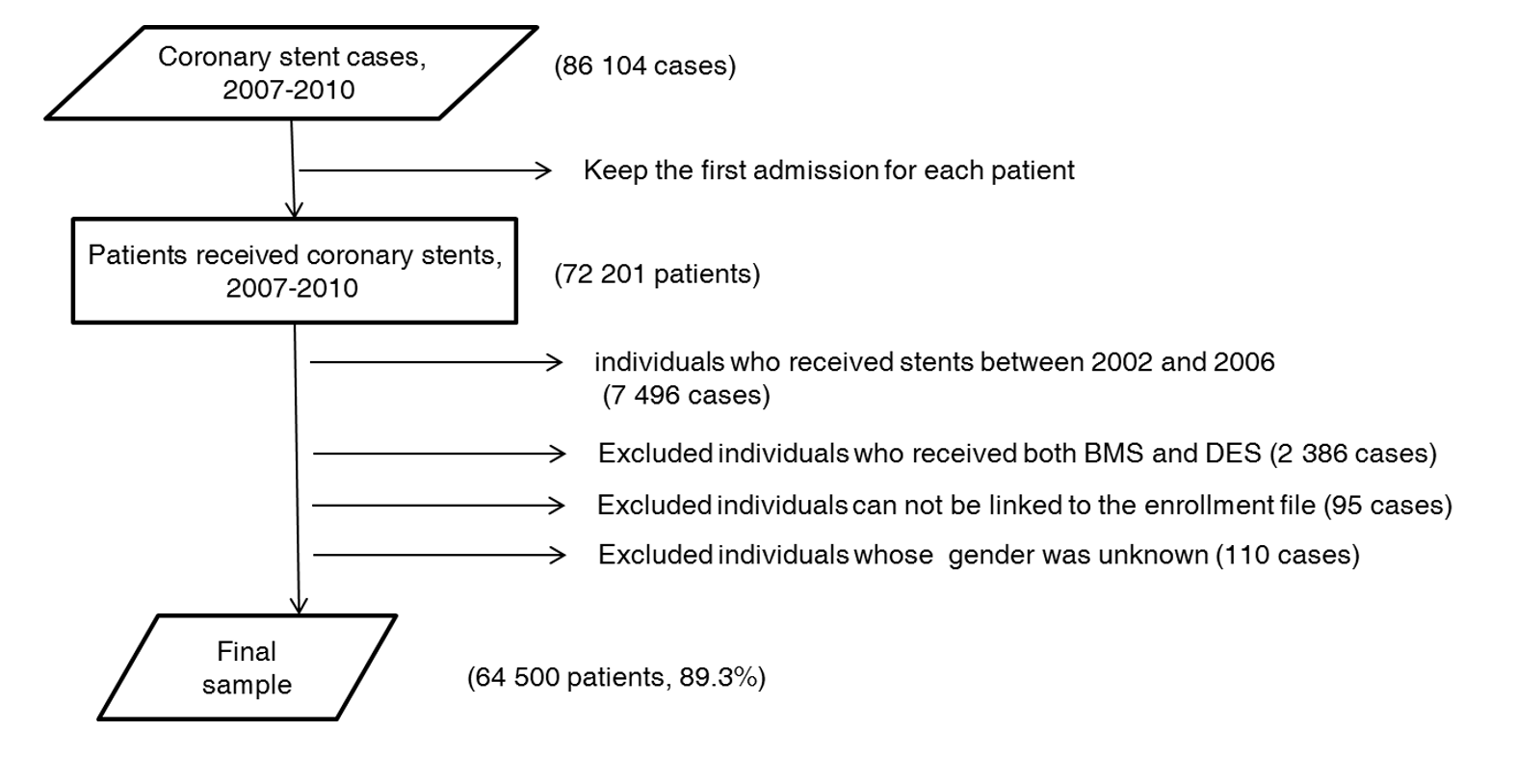

Supplement: S1 Fig — (TIF) [file pone.0179127.s001.tif]
